# Internal and External Training Load in Under-19 versus Professional Soccer Players during the In-Season Period

**DOI:** 10.3390/ijerph18020558

**Published:** 2021-01-11

**Authors:** Sullivan Coppalle, Guillaume Ravé, Jason Moran, Iyed Salhi, Abderraouf Ben Abderrahman, Sghaeir Zouita, Urs Granacher, Hassane Zouhal

**Affiliations:** 1 Laboratoire Mouvement, Sport, Santé, University of Rennes, M2S—EA 1274, F-35000 Rennes, France; sullivan.coppalle@hotmail.fr; 2 Stade Lavallois Mayenne Football Club, 53000 Laval, France; rave.guillaumme@gmail.com; 3 School of Sport, Rehabilitation and Exercise Sciences, University of Essex, Colchester 62326, UK; jmorana@essex.ac.uk; 4 ISSEP Ksar-Essaid, University of La Manouba, Tunis 2000, Tunisia; iyedsalhi38@gmail.com (I.S.); benabderrahmanabderraouf@yahoo.fr (A.B.A.); zouita2003@yahoo.fr (S.Z.); 5 Division of Training and Movement Sciences, University of Potsdam, 14469 Potsdam, Germany

**Keywords:** monitoring, global positioning system, elite athletes, academy, RPE

## Abstract

This study aimed to compare the training load of a professional under-19 soccer team (U-19) to that of an elite adult team (EAT), from the same club, during the in-season period. Thirty-nine healthy soccer players were involved (EAT [*n* = 20]; U-19 [*n* = 19]) in the study which spanned four weeks. Training load (TL) was monitored as external TL, using a global positioning system (GPS), and internal TL, using a rating of perceived exertion (RPE). TL data were recorded after each training session. During soccer matches, players’ RPEs were recorded. The internal TL was quantified daily by means of the session rating of perceived exertion (session-RPE) using Borg’s 0–10 scale. For GPS data, the selected running speed intensities (over 0.5 s time intervals) were 12–15.9 km/h; 16–19.9 km/h; 20–24.9 km/h; >25 km/h (sprint). Distances covered between 16 and 19.9 km/h, > 20 km/h and >25 km/h were significantly higher in U-19 compared to EAT over the course of the study (*p* = 0.023, d = 0.243, small; *p* = 0.016, d = 0.298, small; and *p* = 0.001, d = 0.564, small, respectively). EAT players performed significantly fewer sprints per week compared to U-19 players (*p* = 0.002, d = 0.526, small). RPE was significantly higher in U-19 compared to EAT (*p* = 0.001, d = 0.188, trivial). The external and internal measures of TL were significantly higher in the U-19 group compared to the EAT soccer players. In conclusion, the results obtained show that the training load is greater in U19 compared to EAT.

## 1. Introduction

Playing soccer is associated with considerable physiological stress that leads to fatigue. There is evidence that the presence of physical fatigue is related to an increased risk of sustaining injuries [1,2]. To limit these negative consequences, monitoring players’ training load (TL) is essential to programming, as well as adapting training and recovery processes [3,4]. Malone et al. [5] defined TL as the combination of factors, such as volume and intensity, that can be manipulated in the formulation of training, a concept which comprises of two key components: external and internal load. The applied quantity of the various types of soccer-related activities (training and match play) represent the external load which is defined as the physical work prescribed by coaches [6]. The internal load can be defined as the relative biological stresses imposed on players during training and competition [4].

Using valid and reliable tools is essential in measuring TL [7]. To monitor the internal TL, different parameters like heart rate and blood lactate can be assessed [5,8,9,10]. Perhaps the easiest method to apply is that proposed by Foster et al. [11] who advocated the use of the “Rating of Perceived Exertion” (RPE). This method requires players to subjectively rate the TL of the entire training session using the Foster 0–10 scale [11]. This subjective value is multiplied by the total duration (minutes) of the training session which facilitates the calculation of total stress in arbitrary units (AU) [12]. To monitor external TL, global positioning systems (GPS) and accelerometer-derived parameters are largely used in professional soccer [13,14]. This method allows the monitoring of total distance traversed and distances covered at various speeds, as well as the classification of sprints, accelerations and decelerations [15,16,17].

The RPE and GPS monitoring methods have previously been shown to assist coaches in reducing injury rates in team sport athletes [18]. Recently, Malone et al. [19] reported statistically significant associations between higher weekly training load changes (≥75%) and increased likelihood of sustaining injuries in team sports athletes. It is well known that the physical and physiological demands of soccer matches can vary substantially according to competitive age categories [20,21]. High-level soccer players need to be prepared to respond to sizeable inter-game variability in physical and physiological demands [13,22] and it, therefore, stands to reason that developmental players must also be able to meet such challenges when they graduate to the senior level. One of the most important stages of a player’s soccer development is this transition from academy to the adult team level [23] with the period (i.e., 17–21 years) said to be critical in determining a player’s future career trajectory [23]. However, it is not known if young professional players, who graduate to higher levels of the sport, do so having developed their physical fitness to the required level to compete in the first team of their club [23]. Several professional soccer teams have promoted young players (17–19 years) to the adult team both for training and match scenarios but, despite this, it is difficult to determine if young players possess the physical capacities to sustain the high TLs experienced at the adult levels of the professional game.

Soccer academies’ training processes must conform to the principle of progressivity according to the development of the various constituent age groups within that academy [24]. When a player competes in an U-19 team, it is arguable that he is likely to join an adult team at some stage in the future. However, it has been shown that more experienced players have better technical and tactical skills than younger players and, therefore, exhibit better control of their physical efforts during a match [24]. Because players must be physically prepared to ascend to a higher level, training objectives must be aligned to those of the adult team prior to any graduation. Exemplifying this, Buchheit et al. [20] showed that increases in high and very high intensity running volumes were related to the respective age category. The goal of coaches during the training of young players is, therefore, to develop the young player’s physical capacity such that they can be technically effective during match-play scenarios at the adult level [3].

When a young soccer player graduates to the adult team in his club, coaches must consider whether or not they are prepared to sustain higher training loads without risking serious injury. To our knowledge, only one recent study [25] compared the TL between first and under 19 teams (U-19) during a professional in-season period. In this Dutch study, the authors showed that the total distance covered during training was greater for the U19 team but that this was not the case for distances traversed at a higher intensity [25]. However, in this study only external TL was taken into account and it is well established that internal TL (RPE) represents an important tool to measure the impacts of external TL on the player [26]. In a recent systematic review, Fox et al. [27] concluded that internal TL showed a stronger association with performance than external TL in team sports. Furthermore, TL may differ between soccer academies in Europe depending on the objectives and the demands of a particular national league [28,29]. Given that these factors remain unaddressed in the literature, the purpose of this study was to quantify TL during the in-season period in U19 versus EAT players within the same soccer club and to determine whether the young players experienced TLs comparable to those in the older age category. Based on relevant literature data [20,26,27,28,29], we hypothesised that TL of the U19 group should be comparable to that of the elite adult group.

## 2. Materials and Methods

### 2.1. Participants

A convenience sample of 20 adult elite (age: 25.9 ± 5.2 years; height: 179.0 ± 5.6 cm; mass: 76.4 ± 5.6 kg) and 19 young (age: 18.7 ± 1.3 years; height: 179.5 ± 6.9 cm; mass: 72.7 ± 4.8 kg) soccer players from the same club were enrolled in this study. The EAT were participating in Ligue 2 of the French championship, the second-highest level of soccer in France. The U19 team participated in the U19 National Championship, the highest level in that age category, nationally. All players were notified of the research protocol, benefits and risks before providing written informed consent for participation. The protocol was conducted in accordance with the Declaration of Helsinki and was fully approved by the medical staff of the club and the ethics committee of the University of Rennes 2.

### 2.2. Procedures

To compare the TL of the two teams, a non-interventional study was designed (i.e., no intervention during training). Data were collected during a 4-week period in the second part of the in-season stage during the 2015/2016 season. The periods observed for each group had to include one game per week for each week, without any break in players’ usual activity. Internal (RPE) and external (GPS) training load were recorded after each training session. During matches, only the RPE was recorded. Several types of sessions were excluded from the analysis including individual training, recovery sessions and rehabilitation training. The weeks in which the players were injured were not taken into account for the calculation of training load. 

### 2.3. Training Program

Throughout the study, the two groups trained separately. The EAT team performed an average of five training sessions and one match per week. The U-19 team performed, on average, six training sessions and one match per week. All collective sessions were recorded and used for the analysis Each team also performed two strength training sessions per week. The strength training session consisted of four to six eccentric exercises of the lower limbs with a similar load (series, repetitions, additional load) used across the four weeks. These sessions aimed to develop eccentric strength and muscle power. The other sessions were based on soccer-specific technical and tactical training (Table 1). 

The total number of training sessions was similar between both groups over the four-week period. EAT players, on average, accumulated more matches and minutes played in matches compared to U19 players (Table 2). This is explained by the fact that EAT players who were not playing with the adult team, appeared in the reserve team to maintain fitness. All EAT players experienced extensive playing time in contrast to unselected U19 players who did not undergo additional playing time to address a deficit in playing time.

### 2.4. Quantification of Training Load

#### 2.4.1. Internal TL (Rating of Perceived Exertion [RPE])

The TL was quantified daily using the session rating of perceived exertion (s-RPE) using Borg’s 0–10 scale [11], with “10” representing the most intense level, and “0” the least. Data were collected 15–20 min after each training session. Players from both teams were familiarised with the Borg scale since at least, the beginning of the season in question. The club’s strength and conditioning coach verified each player’s answers for accuracy. The s-RPE was calculated by multiplying the training session duration (minutes) by session RPE according to Foster et al. [11]. It was then measured and presented in arbitrary units (AU). 

#### 2.4.2. External TL (Global Positioning System [GPS])

Distances covered at different intensities were collected daily using GPS technology (GPSPORTS, SPI Pro X 15 Hz, Canberra, Australia). This type of system has previously been shown to provide valid and reliable estimates of movement velocity during acceleration, deceleration, and constant-velocity movements in linear, multidirectional, and soccer-specific activities [30]. The minimum acceptable number of available satellite signals was 8 (range 8–11). The selected running speed intensities (over a 0.5 s time interval) were 12–15.9 km/h; 16–19.9 km/h; >20–24.9 km/h [14]; >25 km/h (sprint). The number of sprints (>25 km/h) and acceleration distances (>2.5m/s^2^) were also analyzed. Players used the same individual GPS units throughout the experimental period to avoid measurement error. These transmitters were started 15-min before training and in an open area to allow satellite connection. They were installed affixed to players just prior to each training session and were removed immediately afterwards. Data were analysed just after each training session.

### 2.5. Statistical Analyses

Data are presented as means and standard deviations (SD). Normality of data distribution was assessed and confirmed using the Shapiro-Wilk test. For statistical analyses, an analysis of covariance (ANCOVA) was computed with the group as a between-subject comparator (U19 and EAT) and baseline data as a covariate. Table 3 illustrates that almost all baseline measures (week 1) were significantly different between U19 and EAT, except for RPE. Due to these baseline differences, ANCOVA and not ANOVA was applied. This method (e.g., ANCOVA) has been proposed as the most sufficient statistical approach for the analysis of continuous outcomes [31]. The analysis comparing the outcomes of EAT versus U19 over the four weeks observation period comprised mean values for the absolute external and internal chronic load. Effect sizes (ES) were calculated from ANCOVA output by converting partial eta-squared to Cohen’s d. Within-group ESs were obtained using the equation ES = (mean post–mean pre)/SD. Effect sizes of 0.20–0.60, 0.61–1.19 and ≥1.20 were considered as small, moderate and large, respectively [32]. In general, descriptive data were presented as baseline adjusted group mean values and standard deviations. A value of *p* < 0.05 was accepted as the minimal level of statistical significance. All analyses were performed using SPSS for Windows, version 16.0; SPSS Inc (Chicago, IL, USA).

## 3. Results

In total, four weeks of training in both U19 and EAT were studied. The weeks when the players did not fully participate in the group sessions were excluded from the study. ANCOVA was carried out for all the tests because there was a significant difference in Week 1 data between both groups, except for RPE (Table 3).

Statistical differences between the absolute external and internal chronic load (the average over four weeks) were found between the two groups (Table 3). No significant difference was observed concerning the distance covered at 12–15.9 km/h (*p* = 0.6, ES = 0.072, trivial) between the two teams. However, compared to EAT, U-19 players covered longer distances between 16 and 20 km/h (*p* = 0.023, ES = 0.243, small), >20 km/h (*p* = 0.016, ES = 0.298, small) and >2.5 m/s^2^ (*p* = 0.001, ES = 0.564, small). Moreover, EAT performed a significantly smaller number of sprints per week compared to U-19 (*p* = 0.002, ES = 0.526, small). The RPE was significantly higher in U-19 compared to EAT players during the study (*p* = 0.001, ES = 0.188, trivial).

## 4. Discussion

This study aimed to compare the in-season TLs of the U-19 team and the EAT in the same professional soccer club. To our knowledge, there are very few studies that evaluate and compare both internal and external TL parameters between two age-differentiated player groups from the same soccer club and such an investigation is required to evaluate the challenge imposed on young players graduating to their club’s first-team squad.

In our study, the weekly internal TL in the EAT team was similar to that in a previous investigation in a Korean professional soccer team [33]. Moreover, the weekly internal TL in the U-19 team (1588.6 AU) was lower than that of other U19 teams reported by Impellizzeri et al. [34] and Raya-Gonzalez et al. [28] (2605 and 2664 AU, respectively) during the same period of the season. The observed intensity of the evaluated training sessions seems to represent the only significant point of variation between these studies and our study. On this, it is plausible that the playing philosophy of the studied teams varied according to the specific country in which the data were collected thus impacting the intensity of the sessions and matches that were observed. 

One of the main results of our study was that TL was higher in the U-19 team than in the EAT team both for the internal (i.e., RPE) and external measures (i.e., distances: 16–19.9 km/h and >20 km/h, accelerations >2.5 m/s^2^ and the number of sprints). These results are contradictory to those reported in a previous study [28] which showed a lower external load in an U-19 team as compared to an EAT. A possible explanation for this is that an U-19 team could have a higher absolute weekly TL given the developmental nature of the playing level and an increased emphasis on fitness training. In this way, the respective levels of soccer could differ in that an EAT may be more focused on winning games and recovering between those games than they are in maintaining physical fitness throughout a season [35].

Despite the gap in TL observed between the EAT and U19 players in this study, the conclusion that this occurred because the younger group trained at a higher volume and intensity does not fully explain the reasons for the observed differences. To thrive, young players must learn to think abstractly and to foster sport-specific strategic behaviour on the field of play during training and match situations [36]. It is plausible that this function is better developed in more experienced players as it is thought that pattern recognition in soccer develops as a player gains in expertise [37]. This is demonstrated by elite players’ generally superior technical skills, as well as their ability to process information from multiple sources [24]. Accordingly, the higher running volumes and speeds seen in the younger players in this study could be representative of a greater dependence on the physical and physiological elements of soccer play, whereby a technically inferior younger player must bridge the skill-gap to the higher level by engaging in a higher volume of on-field activity such as sprinting. Conversely, a tactically well-versed older player could theoretically be less reliant on such playing behaviour, using superior strategic awareness and anticipatory skills to inform their decision making, thus reducing overall movement, but increasing on-field efficiency and effectiveness.

The present findings tentatively demonstrate that the varying objectives of different teams within the same club can influence the TL that the players in those teams are exposed to. In the current economic context, there is an increased emphasis on the promotion of young players to compete in a club’s first team. This can be a cost-saving exercise for a team which lacks the financial resources to sign experienced players to play at the highest level. The present findings demonstrate that from an athletic perspective, U19 players seem well prepared to sustain high TLs and to operate alongside EAT players if required to. To facilitate a smooth transition for the developmental player into a club’s adult team, coaches need to ensure that players are exposed to sufficient TLs to ensure that they are physically prepared to play at a higher level. Therefore, EAT coaches must monitor the TL of the various age-grade teams of a club to avoid too high an increase in the demands placed on players who graduate from academy level to the EAT. However, it is also necessary to address other factors such as the technical skill and tactical learning which differentiate the two age categories. This can create substantial challenges for players as they are introduced to adult-level soccer. Future research is needed to verify the relationships between injuries, training and match loads over the longer term for players who graduate from academies to adult soccer.

Several limitations should be acknowledged in the current study: (1) The impact of match-play could not be studied in conjunction with the data collected for the weekly external TL. Thorpe et al. [35] showed that a match is a weekly activity with the highest load and this is, therefore, important to also consider. (2) Due to time constraints in following up on the collected data, the duration of the study was only four weeks in duration. Most similar studies adopt an observation period of between six weeks and two seasons [5,18,29,38,39]. However, shorter studies do also exist, one example being that of Thorpe et al. [40] who observed the effects of the TL over a 17-day period.

## 5. Conclusions

During the observation period of this study, significant differences were observed between the U19 team and EAT with the younger players demonstrating larger internal and external TLs. With reference to our findings, young French players could join their professional team’s adult training group without experiencing a deleterious increase in TL. It must, however, be indicated that this may differ between teams, leagues and countries. Before a young player (e.g., U19 or U17) joins the senior team of his club, coaches need to verify if the TL that is applied in the academy is preparative for that which would be experienced at the higher level. This may assist the young player in avoiding fatigue and injury as they graduate from academy to professional soccer.

## Figures and Tables

**Table 1:** Weekly training organization for both groups.

*TEAM*		Monday	Tuesday	Wednesday	Thursday	Friday	Saturday	Sunday
EAT	AM	Strength session + Technical soccer session	Strength session + Technical and physical soccer session	Technical soccer session	Tactical soccer session	Rest	Recovery training session	Rest
	PM	Rest	Rest	Rest	Rest	Match	Rest	Rest
U-19	AM	Rest	Rest	Strength session + Technical soccer session	Rest	Rest	Rest	
	PM	Rest	Technical soccer session	Tactical and physical soccer session	Technical soccer session	Tactical soccer session	Match	Recovery training session

ADU: Professional players from an elite team, U-19: Under 19 years old.

**Table 2:** Summary of match and training volume for both groups.

*TEAM*	Total Number of Sessions	Total Number of Matches	Total Number of Minutes Played in Match (min)
EAT	16.5 (±2.5)	3.1 (±0.8)	245 (±88.4)
U-19	16.4 (±3.1)	1.9 (±0.8)	143 (±71.3)

EAT: Professional players from an elite team, U-19: Under 19 years old.

**Table 3:** Internal and external training load of U-19 and professional teams over four weeks.

Parameters	EAT Players					U19 Players					*p* (Cohen’s d)
	Week 1	Week 2	Week 3	Week 4	Means (±SD)	Week 1	Week 2	Week 3	Week 4	Means (±SD)	
D 12–15.9 (km/h)	2076.9 $	2329.6	2866.9	2469.2	2435.6 ± 842.6	2647.3	2789.4	2670.2	2981.5	2772.1 ± 574.3	0.6 (0.072)
D 16–19.9 (km/h)	739.2 $	797.2	845.2	912.2	823.4 ± 203.2	991.0	1105.8	1596.6	1384	1269.3 ± 419.7	0.023 * (0.243)
D > 20 (km/h)	329.9 $	223.2	472.4	476.8	375.0 ± 235.7	639.0	532.8	1065.2	731	742.0 ± 337.4	0.016 * (0.298)
D > 2.5 m/s^2^	1412.2 $	1256.6	1493.6	1624.2	1446.6 ± 64.5	1930.6	2084.9	2977.4	2212	2301.2 ± 749.2	0.001 * (0.564)
Number of sprints	66.8 $	58.4	56.6	68.8	62.6 ± 27.3	86.6	87.4	157	86	104.2 ± 44.7	0.002 * (0.526)
RPE (AU)	1691.1	1442.1	1479.3	1439.7	1515.6 ± 207.0	1706.9	1815.4	1476.3	1309.3	1588.6 ± 311.7	0.001 * (0.188)

*: significant differences between means for EAT and U-19. $: significant differences between values of week 1 for ELT and U-19. D: Distance.

## Data Availability

Data are available upon request to corresponding author.
